# Association of platelet-to-lymphocyte ratio and neutrophil-to-lymphocyte ratio with outcomes in stroke patients achieving successful recanalization by endovascular thrombectomy

**DOI:** 10.3389/fneur.2022.1039060

**Published:** 2022-12-16

**Authors:** Jin Ma, Wenting Guo, Jiali Xu, Sijie Li, Changhong Ren, Longfei Wu, Chuanjie Wu, Chuanhui Li, Jian Chen, Jiangang Duan, Qingfeng Ma, Haiqing Song, Wenbo Zhao, Xunming Ji

**Affiliations:** ^1^Department of Neurology, Xuanwu Hospital, Capital Medical University, Beijing, China; ^2^Beijing Key Laboratory of Hypoxic Conditioning Translational Medicine, Xuanwu Hospital, Capital Medical University, Beijing, China; ^3^Department of Emergency, Xuanwu Hospital, Capital Medical University, Beijing, China; ^4^Department of Neurosurgery, Xuanwu Hospital, Capital Medical University, Beijing, China; ^5^Beijing Institute of Brain Disorders, Laboratory of Brain Disorders, Ministry of Science and Technology, Collaborative Innovation Center for Brain Disorders, Capital Medical University, Beijing, China

**Keywords:** acute ischemic stroke, platelet-to-lymphocyte ratio, neutrophil-to-lymphocyte ratio, monocyte-to-lymphocyte ratio, endovascular thrombectomy

## Abstract

**Objective:**

Serum inflammatory biomarkers play crucial roles in the development of acute ischemic stroke (AIS). In this study, we explored the association between inflammatory biomarkers including platelet-to-lymphocyte ratio (PLR), neutrophil-to-lymphocyte ratio (NLR), and monocyte-to-lymphocyte ratio (MLR), and clinical outcomes in AIS patients who achieved successful recanalization.

**Methods:**

Patients with AIS who underwent endovascular thrombectomy (EVT) and achieved a modified thrombolysis in the cerebral infarction scale of 2b or 3 were screened from a prospective cohort at our institution between January 2013 and June 2021. Data on blood parameters and other baseline characteristics were collected. The functional outcome was an unfavorable outcome defined by a modified Rankin Scale of 3–6 at the 3-month follow up. Other clinical outcomes included symptomatic intracranial hemorrhage (sICH) and 3-month mortality. Multivariable logistic regression analysis was performed to evaluate the effects of PLR, NLR, and MLR on clinical outcomes.

**Results:**

A total of 796 patients were enrolled, of which 89 (11.2%) developed sICH, 465 (58.4%) had unfavorable outcomes at 3 months, and 168 (12.1%) died at the 3-month follow up. After adjusting for confounding variables, a higher NLR (OR, 1.076; 95% confidence interval [CI], 1.037–1.117; *p* < 0.001) and PLR (OR, 1.001; 95%CI, 1.000–1.003; *p* = 0.045) were significantly associated with unfavorable outcomes, the area under the receiver operating characteristic curve of NLR and PLR was 0.622 and 0.564, respectively. However, NLR, PLR, and MLR were not independently associated with sICH and 3-month mortality (all adjusted *p* > 0.05).

**Conclusion:**

Overall, our results indicate that higher PLR and NLR were independently associated with unfavorable functional outcomes in AIS patients with successful recanalization after EVT; however, the underlying mechanisms are yet to be elucidated.

## Background

Previous randomized controlled trials have demonstrated that patients with acute ischemic stroke (AIS) secondary to large vessel occlusion could benefit from reperfusion therapy with endovascular thrombectomy (EVT) (1, 2). However, approximately half of patients who achieve successful recanalization of the occluded artery post-EVT have unfavorable outcomes at 90 days (3–5). The mechanisms underlying the mismatch of successful recanalization and good outcomes remain unclear (6).

The neuroinflammatory response has been increasingly recognized to be important in the pathophysiology of AIS (7). Activation of leukocytes, platelets, or other pro-inflammatory mediators plays a vital role in AIS neurological prognoses. The potential novel biomarkers of inflammation, platelet-to-lymphocyte ratio (PLR), neutrophil-to-lymphocyte ratio (NLR), and monocyte-to-lymphocyte ratio (MLR), have recently been proposed as critical predictors of unfavorable outcomes in patients with AIS (8, 9). It has been found that NLR and PLR in AIS patients with a National Institutes of Health Stroke Scale (NIHSS) ≥6 were significantly higher than in patients with a NIHSS <6, indicating the severity of stroke was related to the value of NLR and PLR (10). In addition, higher NLR and MLR have been found to be positively correlated with stroke severity, adverse complications, and death (11, 12), while higher PLR predicted unfavorable functional outcomes with a higher modified Rankin Scale (mRS) and NIHSS scores (13). However, few studies support the predictive value of NLR, PLR, and MLR on clinical outcomes in AIS patients with successful recanalization (14). In this study we aimed to explore the association of PLR, NLR, and MLR with clinical outcomes in patients with AIS who underwent EVT and achieved successful recanalization.

## Methods

### Study design

Data for this study were obtained from a prospective cohort of consecutive patients with AIS who underwent EVT at our hospital between January 2013 and June 2021. Information on the prospective cohort, EVT procedure for AIS, and imaging evaluations have been described previously (15). This study was approved by the Ethics Committee of Xuanwu Hospital, and written informed consent was obtained from all patients or their legally authorized representatives.

### Study population

The inclusion criteria for this study were as follows: (1) age ≥18 years, (2) treatment with EVT within 24 h and successful recanalization, defined as a modified Thrombolysis in Cerebral Infarction (mTICI) of 2b or 3. The exclusion criteria were as follows: (1) pre-stroke mRS > 2, (2) absence of blood parameters before EVT, and (3) lack of 3-month follow-up.

### Data collection

Variables including demographics, vascular risk factors, baseline clinical assessment (admission systolic blood pressure [SBP], diastolic blood pressure [DBP], NIHSS, Alberta Stroke Program Early Computed Tomography Score [ASPECTS], or posterior circulation Alberta Stroke Program Early Computed Tomography Score [pc-ASPECTS]), laboratory tests (fasting blood glucose [FBG], NLR, PLR, MLR), lesion location, stroke etiology, treatment (general anesthesia, time interval from symptom onset to puncture [OTP], time interval from symptom onset to recanalization [OTR], intravenous thrombolysis [IVT]), intracranial hemorrhage (ICH), symptomatic intracranial hemorrhage (sICH), and clinical outcomes at 3 months were collected from the database and analyzed.

### Assessment of NLR, PLR, and MLR

Blood samples were collected within 10 min of arrival at the hospital. Parameters including neutrophils, lymphocytes, monocytes, and platelets were analyzed using an automated blood cell counter (MEK-722K, NIHON, KOHEN, JAPAN). The NLR, PLR, and MLR were calculated by dividing the number of neutrophils, platelets, and monocytes by the number of lymphocytes.

### Assessment of clinical outcomes

The functional outcome was an unfavorable outcome at 3 months defined as an mRS of 3–6 (16). Other clinical outcomes were sICH and mortality at the 3-month follow up. The sICH was diagnosed according to the European Cooperative Acute Stroke Study III (17) as ICH associated with any of the following conditions: (1) NIHSS score increased >4 points; (2) clinical deterioration determined by investigators, or adverse events including drowsiness and increase of hemiparesis (18, 19).

### Statistical analyses

All enrolled patients were divided into favorable and unfavorable outcome groups according to their 3 months mRS score as previously described. Differences in baseline characteristics between the two groups were analyzed. Continuous variables were expressed as mean ± standard deviation (SD) or median (interquartile range, IQR). Analysis was performed using the *t*-test for independent samples or the Mann-Whitney *U*-test, respectively. Categorical variables were described as numbers (percentages) and analyzed using the chi-square test. Multivariable logistic regression analysis was performed to explore the effect of NLR, PLR, and MLR on 3-month functional outcomes, adjusting for age, sex, diabetes, hyperlipidemia, atrial fibrillation, admission DBP, NIHSS, ASPECTS, FBG, lesion location, general anesthesia, and sICH. Receiver operating characteristic (ROC) curves were used to test the discriminative ability of the NLR, MLR, and PLR for 3-month functional outcomes. In addition, the association between NLR, MLR, PLR, and sICH as well as mortality at 3 months was also analyzed.

Statistical analyses were performed using SPSS statistical software (version.26; IBM Corp., Armonk, NY, USA). Statistical significance was indicated by *p* < 0.05.

## Results

**Figure 1:**
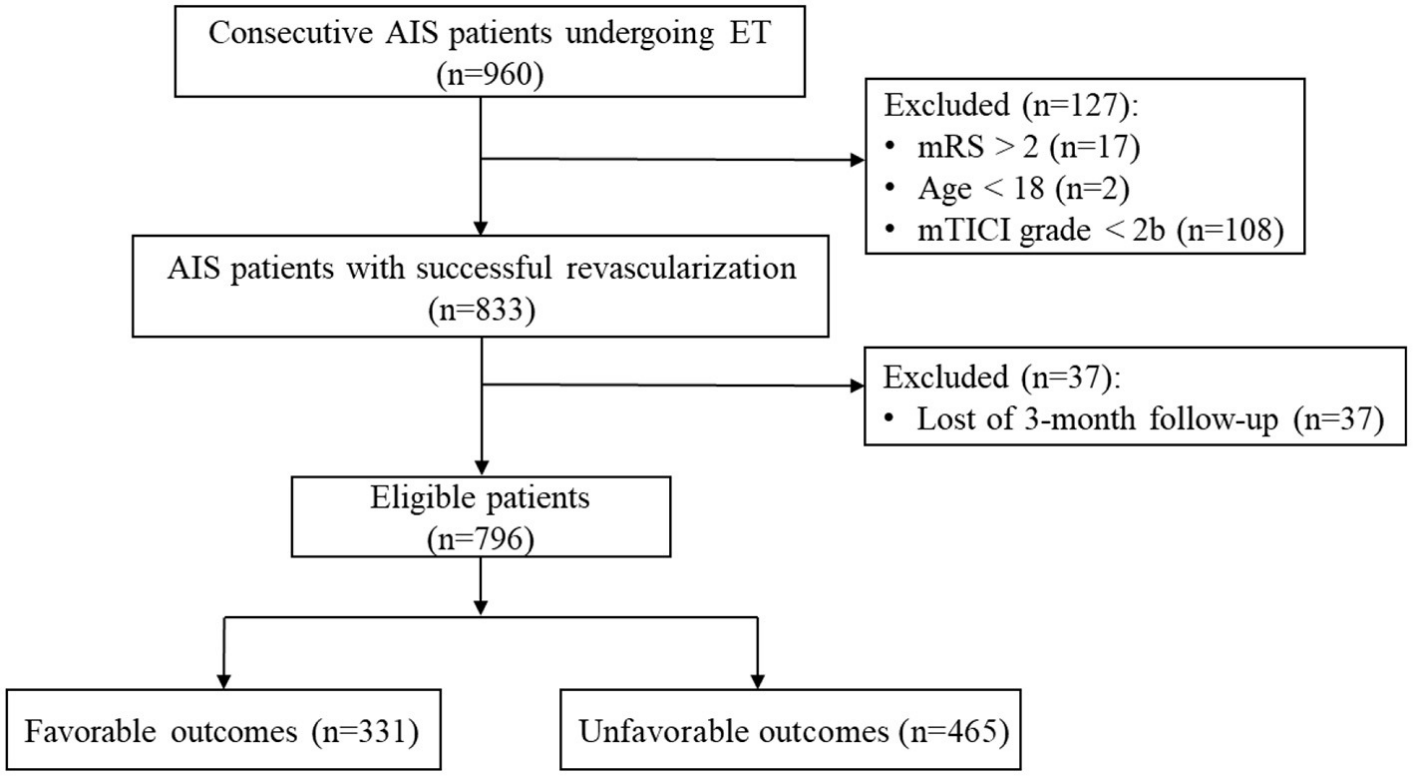
Study flow chart. AIS, acute ischemic stroke; EVT, endovascular thrombectomy; mRS, modified Rankin Scale; mTICI, modified Thrombolysis in Cerebral Infarction.

A total of 960 patients with AIS who underwent EVT were screened, and 796 patients who fulfilled the inclusion criteria were included in the study (Figure 1). The mean age of the patients was 62.89 ± 12.22 years, and 566 (71.1%) were male. The median baseline NIHSS and ASPECTS/pc-ASPECTS scores were 16 and 9, respectively. Large-vessel occlusion in the anterior circulation was observed in 568 patients (71.4 %). A total of 270 patients (33.9%) underwent IVT before EVT. The median OTP and OTR were 380 and 458 min, respectively. sICH occurred in 89 (11.2%) patients. During the follow-up at 3 months, 465 (58.4%) patients had unfavorable functional outcomes and 168 (12.1%) patients died.

### Univariate analyses of patients with favorable and unfavorable outcomes

A comparison of the detailed characteristics of the patients with favorable and unfavorable outcomes is shown in Table 1. In the univariable analysis, patients with unfavorable outcomes were much older (65.07 ± 11.93 vs. 59.83 ± 11.98, *p* < 0.001), had higher proportions of diabetes (34.0 vs. 21.1%, *p* < 0.001), hyperlipidemia (69.5 vs. 42.0%, *p* < 0.001), previous stroke (29.5 vs. 20.2%, *p* = 0.003), posterior circulation lesion (32.9 vs. 22.7%, *p* = 0.002), general anesthesia (42.2 vs. 29.9%, *p* < 0.001), ICH (44.1 vs. 22.7%, *p* < 0.001), and sICH (17.8 vs. 1.8%, *p* < 0.001). However, there was a lower proportion of current smokers (35.7 vs. 45.6%, *p* = 0.005).

**Table 1:** Characteristics of patients with favorable and unfavorable outcomes.

**Factors**	**Total number** ** (*n* = 796)**	**Favorable outcomes** ** (*n* = 331)**	**Unfavorable outcomes** ** (*n* = 465)**	***P*-value**
**Demographics**			
Age, y, mean ± SD	62.89 ± 12.22	59.83 ± 11.98	65.07 ± 11.93	<0.001^*^
Male, *n* (%)	566 (71.1%)	256 (77.3%)	310 (66.7%)	<0.001^*^
**Vascular risk factors**			
Hypertension, *n* (%)	558 (70.1%)	220 (66.5%)	338 (72.7%)	0.059
Diabetes, *n* (%)	228 (28.6%)	70 (21.1%)	158 (34.0%)	<0.001^*^
Hyperlipidemia, *n* (%)	462 (58.0%)	139 (42.0%)	323 (69.5%)	<0.001^*^
Current smoking, *n* (%)	317 (39.8%)	151 (45.6%)	166 (35.7%)	0.005^*^
Atrial fibrillation, *n* (%)	259 (32.5%)	95 (28.7%)	164 (35.3%)	0.051
Previous stroke, *n* (%)	204 (25.6%)	67 (20.2%)	137 (29.5%)	0.003^*^
**Baseline clinical assessment**			
Admission SBP (mmHg), mean ± SD	146 ± 33	143.55 ± 22.58	150.67 ± 24.15	<0.001^*^
Admission DBP (mmHg), mean ± SD	83 ± 15	83.03 ± 14.43	85.27 ± 14.79	0.033^*^
Admission NIHSS, median (IQR)	16 (12–21)	13 (10–17)	18 (14–26)	<0.001^*^
Admission ASPECTS/pc-ASPECTS, median (IQR)	9 (7–10)	9 (8–10)	8 (7–10)	0.006^*^
**Laboratory test**			
FBG (mmol/L), median (IQR)	7.37 (6.15–9.49)	6.89 (5.71–8.25)	7.96 (6.56–10.48)	<0.001^*^
NLR, median (IQR)	5.87 (3.39–9.82)	4.85 (2.79–7.76)	6.57 (4.00–11.21)	<0.001^*^
PLR, median (IQR)	161.79 (108.46–241.67)	153.90 (105.04–218.23)	168.89 (114.38–254.48)	0.002^*^
MLR, median (IQR)	0.30 (0.22–0.42)	0.28 (0.21–0.37)	0.32 (0.22–0.47)	<0.001^*^
**Lesion location**				0.002^*^
Anterior circulation, *n* (%)	568 (71.4%)	256 (77.3%)	312 (67.1%)	
Posterior circulation, *n* (%)	228 (28.6%)	75 (22.7%)	153 (32.9%)	
**Stroke etiology**			
LAA, *n* (%)	479 (60.2%)	210 (63.4%)	269 (57.8%)	0.088
CE, *n* (%)	281 (35.3%)	103 (31.1%)	178 (38.3%)	
Others, *n* (%)	36 (4.5%)	18 (5.4%)	18 (3.9%)	
**Treatment**			
General anesthesia, *n* (%)	295 (37.1%)	99 (29.9%)	196 (42.2%)	<0.001^*^
OTP (min), median (IQR)	380 (284–528)	389 (286–540)	375 (282–520)	0.775
OTR (min), median (IQR)	458 (358–600)	450 (360–597)	465 (353–602)	0.507
IVT, *n* (%)	270 (33.9%)	112 (33.8%)	158 (34.0%)	0.967
**Clinical outcomes**			
ICH, *n* (%)	280 (35.2%)	75 (22.7%)	205 (44.1%)	<0.001^*^
sICH, *n* (%)	89 (11.2%)	6 (1.8%)	83 (17.8%)	<0.001^*^

* *P* < 0.05. SBP, systolic blood pressure; DBP, diastolic blood pressure; NIHSS, National Institute of Health Stroke Scale; ASPECTS, Alberta Stroke Program Early Computed Tomography Score; pc-ASPECTS, posterior circulation Alberta Stroke Program Early Computed Tomography Score; FBG, fast blood glucose; NLR, neutrophil-to-lymphocyte ratio; PLR, platelet-to-lymphocyte ratio; MLR, monocyte-to-lymphocyte ratio; LAA, large artery atherosclerosis; CE, cardio embolism; OTP, time interval from symptoms onset to puncture; OTR, time interval from symptoms onset to recanalization; IVT, intravenous thrombolysis; ICH, intracranial hemorrhage; sICH, symptomatic intracranial hemorrhage.

The results showed that men were more likely to favorable outcomes (77.3 vs. 66.7%, *p* < 0.001). In addition, patients with unfavorable outcomes also had higher baseline SBP (150.67 ± 24.15 vs. 143.55 ± 22.58 mmHg, *p* < 0.001), higher DBP (85.27 ± 14.79 vs. 83.03 ± 14.43 mmHg, *p* = 0.033), higher NIHSS score (median, 18 vs. 13, *p* < 0.001), lower ASPECTS/pc-ASPECTS score (median, 8 vs. 9, *p* = 0.006). For laboratory tests, patients in the unfavorable outcome group had higher FBG (median, 7.96 vs. 6.89 mmol/L, *p* < 0.001), NLR (median, 6.57 vs. 4.85, *p* < 0.001), PLR (median, 168.89 vs. 153.90, *p* = 0.002), and MLR (median, 0.32 vs. 0.28, *p* < 0.001).

### Effect of NLR, PLR, and MLR on 3-month functional outcomes

After adjusting for potential confounders (age, sex, diabetes, hyperlipidemia, atrial fibrillation, admission DBP, NIHSS, ASPECTS, FBG, lesion location, general anesthesia, and sICH), NLR (OR, 1.076; 95% CI, 1.037–1.117; *p* < 0.001), and PLR (OR, 1.001; 95% CI, 1.000–1.003; *p* = 0.045) were found as independent predictors of unfavorable outcomes. Nevertheless, MLR was not significantly associated with unfavorable outcomes (OR, 1.052; 95% CI, 0.954–2.365; *p* = 0.079) (Table 2).

**Table 2:** Multivariable analysis of NLR, PLR, MLR in predicting clinical outcomes.

**Variable**	**β**	**SE**	**Adjusted OR**	**Adjusted 95% CI**		***P*-value**
**Unfavorable outcomes at 3 months**					
NLR^@^	0.074	0.019	1.076	1.037	1.117	< 0.001^*^
PLR^@^	0.001	0.001	1.001	1.000	1.003	0.045^*^
MLR^@^	0.407	0.232	1.502	0.954	2.365	0.079
**sICH**					
NLR^&^	0.010	0.015	1.010	0.980	1.042	0.500
PLR^&^	0.001	0.001	1.000	0.998	1.001	0.601
MLR^&^	0.057	0.265	1.059	0.630	1.778	0.830
**Mortality at 3 months**					
NLR^@^	0.022	0.013	1.023	0.997	1.049	0.082
PLR^@^	0.001	0.001	1.001	1.000	1.002	0.268
MLR^@^	0.193	0.183	1.213	0.847	1.737	0.292

* *P* < 0.05.

@ Adjusting for age, sex, diabetes, hyperlipidemia, atrial fibrillation, admission DBP, NIHSS, ASPECTS/pc-ASPECTS, FBG, lesion location, general anesthesia, and sICH.

& Adjusting for age, admission SBP, FBG, lesion site, TOAST, and IVT.

**Figure 2:**
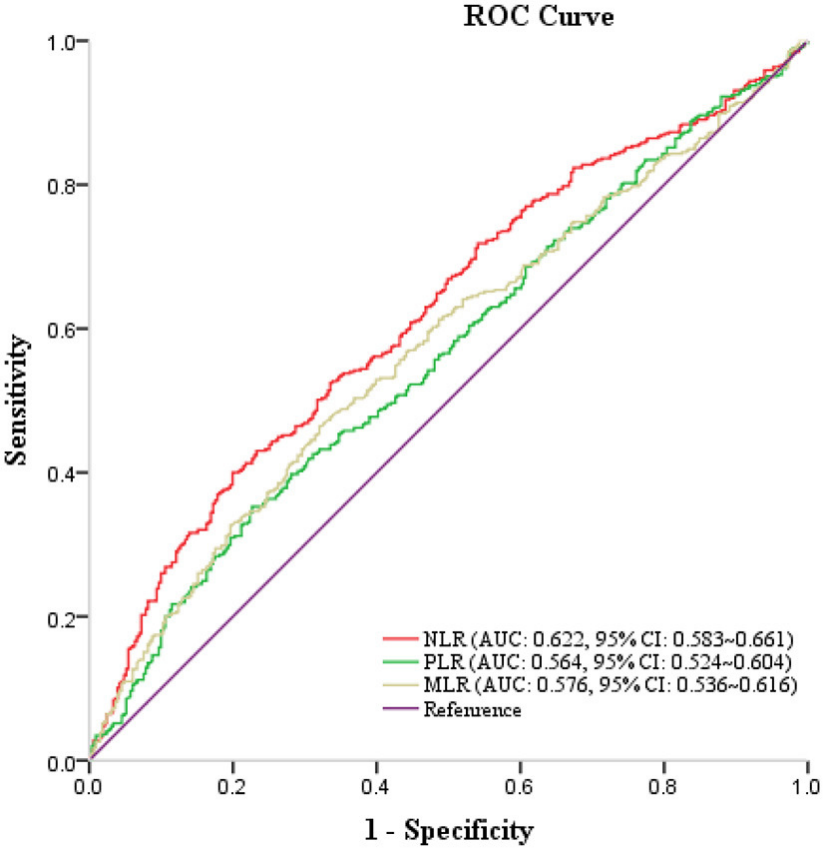
Receiver operating characteristic curve of NLR, PLR, MLR in predicting unfavorable outcomes at 3 months.

The areas under the receiver operating characteristic curves (AUC) of NLR, PLR, and MLR were 0.622 (95% CI, 0.583–0.661; *p* < 0.001), 0.564 (95% CI, 0.524–0.604; *p* = 0.002), and 0.576 (95% CI, 0.536–0.616; *p* < 0.001), respectively (Figure 2).

### Effect of NLR, PLR, and MLR on sICH and 3-month mortality

We also analyzed the relationship between NLR, PLR, MLR, and sICH as well as 3-month mortality. After adjusting for potential confounders, NLR, PLR, and MLR was neither significantly associated with sICH [NLR (OR: 1.010, 95% CI: 0.980–1.042, *p* = 0.500), PLR (OR: 1.000, 95% CI: 0.998–1.001, *p* = 0.601), MLR (OR: 1.059, 95% CI: 0.630–1.778, *p* = 0.830)] nor mortality at 3 months [NLR (OR: 1.023, 95% CI: 0.997–1.049, *p* = 0.082), PLR (OR: 1.001, 95% CI: 1.000–1.002, *p* = 0.268), MLR (OR: 1.213, 95% CI: 0.847–1.737, *p* = 0.292)].

## Discussion

In this study, we found that approximately half (58.4%) of the patients with successful recanalization still had unfavorable outcomes at follow-up after 3 months. Moreover, higher NLR and PLR before EVT were significantly associated with unfavorable functional outcomes in patients with AIS who achieved successful recanalization after EVT.

Currently, EVT is recognized as the most effective reperfusion therapy for the treatment of AIS secondary to the occlusion of large vessels (20). Despite EVT yielding a successful recanalization rate of >80% compared with traditional therapies, around half of the patients who achieved successful recanalization still suffer from unfavorable functional outcomes (3), as was observed in this study. Possible causes include subsequent secondary brain injury from cerebral edema (CED), hemorrhagic transformation, and infarct growth due to impaired microvascular reperfusion mediating early neurological deterioration and 3-month unfavorable functional outcomes (21, 22). The inflammatory response plays an essential role in the pathophysiology and predicting the prognosis of ischemia or hemorrhagic stroke (23, 24). In acute ischemic stroke, the inflammatory response may worsen the CED, ICH, and delay cerebral ischemia thereby leading to poor prognosis (8, 9, 21).

In this study, we found that NLR and PLR before EVT were significantly associated with unfavorable functional outcomes in patients with AIS after successful recanalization with EVT. Firstly, to determine whether these inflammatory indexes increased the prognosis of poor function by increasing sICH, we analyzed the relationship between PLR, NLR, MLR, and sICH. However, no significant association between inflammatory indexes and sICH was found. Secondly, recent imaging studies have shown that the no-reflow phenomenon, which indicates incomplete microvascular reperfusion of the tissue despite successful macrovascular revascularization, provides insights into the underlying mechanisms of this unfavorable prognosis of successfully recanalized stroke (25). However, advanced perfusion imaging for the evaluation of microvascular tissue reperfusion is too time-consuming for timely treatment, thus difficult to implement in the clinic. In patients with acute myocardial infarction treated with percutaneous coronary intervention, composite inflammatory biomarkers have been shown to be strong predictors of both the no-reflow phenomenon and unfavorable functional outcomes (26). We hypothesized that NLR, PLR, and MLR may mediate neurological outcomes through microvascular no-reflow mechanism in patients with AIS treated with EVT. Theoretically, ischemic brain tissues can release various cytokines and chemokines to guide the proliferation and migration of peripheral leukocytes (27). Elevated levels of peripheral leukocytes transmigrating and infiltrating to the ischemic tissues may cause thrombosis, aggravate endothelial edema, and lead to microvascular occlusion, thereby participating in the microvascular no-reflow phenomenon (25). Therefore, composite inflammation indexes such as NLR, PLR, and MLR, are easy-to-acquire biomarkers, which mayserve as potential predictors of the no-reflow phenomenon, and could be associated with unfavorable functional outcomes in successfully recanalized patients with AIS (28, 29). In the present study, NLR and PLR were found to be independently correlated with functional outcome; however, MLR was not significantly associated with functional outcome. Further investigations are needed to explore the relationship between inflammatory indices, the no-reflow phenomenon, and functional outcomes in human ischemic stroke.

In addition, inflammatory biomarkers have been found to predict functional outcomes in patients with intracerebral (23) and subarachnoid hemorrhage (24). Therefore, in either ischemic or hemorrhagic stroke, inflammatory biomarkers may share common mechanisms in mediating secondary brain injury following acute vascular events. Moreover, anti-inflammatory therapy targeting their common pathways may help improve the neurological prognosis of patients with acute stroke (27).

This study had some limitations. Firstly, the cohort included subjects from only one region of China, which would have introduced selection bias. Therefore, further exploration using larger multicenter prospective studies is warranted to substantiate our findings. Second, covariates related to AIS could not be completely collected due to data limitations. Thirdly, the area under the ROC curve values of NLR, PLR, and MLR for outcome prediction were relatively low and need further exploration. Finally, only preoperative inflammatory indicators were evaluated without post-operative indicators; therefore, post-operative inflammatory indicators will need to be evaluated in future studies.

## Conclusion

This study showed that NLR and PLR before EVT were significantly associated with 3-month functional outcomes in patients with AIS who achieved successful recanalization after EVT. Further studies are needed to confirm these results and explore the underlying mechanisms.

## Data availability statement

The original contributions presented in the study are included in the article/supplementary material, further inquiries can be directed to the corresponding authors.

## Ethics statement

The studies involving human participants were reviewed and approved by the Ethics Committee of Xuanwu Hospital. Written informed consent to participate in this study was provided by the patient/participants or patient/participants' legal guardian/next of kin.

## Author contributions

JM and WG conceived of the study idea, collected and analyzed the data, and drafted the manuscript. JX, LW, and WZ participated in the data collection and analysis. XJ, SL, CR, CW, CL, JC, JD, QM, and HS participated in the coordination of the study. WZ and XJ helped to interpret the data and modify the manuscript. All authors read and approved the final manuscript.
